# Fermented Brown Rice and Rice Bran with *Aspergillus oryzae* (FBRA) Prevents Inflammation-Related Carcinogenesis in Mice, through Inhibition of Inflammatory Cell Infiltration

**DOI:** 10.3390/nu7125531

**Published:** 2015-12-08

**Authors:** Kunishige Onuma, Yusuke Kanda, Saori Suzuki Ikeda, Ryuta Sakaki, Takuya Nonomura, Masanobu Kobayashi, Mitsuhiko Osaki, Masataka Shikanai, Hiroshi Kobayashi, Futoshi Okada

**Affiliations:** 1 Division of Pathological Biochemistry, Tottori University Faculty of Medicine, Tottori 683-8503, Japan; k.onuma@med.tottori-u.ac.jp (K.O.); kanda@med.tottori-u.ac.jp (Y.K.); r_sakaki1986@yahoo.co.jp (R.S.); M15M8005Z@edu.tottori-u.ac.jp (T.N.); osamitsu@med.tottori-u.ac.jp (M.O.); 2 Japanese Red Cross Society, Minato-ku, Tokyo 105-8521, Japan; s04313025@yahoo.co.jp; 3 School of Nursing and Social Services, Health Sciences University of Hokkaido, 1757 Kanazawa, Ishikari-Tobetsu, Hokkaido 061-0293, Japan; mkobaya@hoku-iryo-u.ac.jp; 4 Chromosome Engineering Research Center, Tottori University, Tottori 683-8503, Japan; 5 Genmai Koso Co., Ltd., Sapporo, Hokkaido 001-0012, Japan; shikanai@genmaikoso.co.jp; 6 Sapporo Cancer Seminar Foundation, Sapporo, Hokkaido 001-0012, Japan; scs-hk@phoenix-c.or.jp

**Keywords:** fermented brown rice and rice bran with *Aspergillus oryzae* (FBRA), inflammation-related carcinogenesis, inflammation

## Abstract

We have established an inflammation-related carcinogenesis model in mouse, in which regressive QR-32 cells subcutaneously co-implanted with a foreign body—gelatin sponge—convert themselves into lethal tumors due to massive infiltration of inflammatory cells into the sponge. Animals were fed with a diet containing 5% or 10% fermented brown rice and rice bran with *Aspergillus oryzae* (FBRA). In 5% and 10% FBRA diet groups, tumor incidences were lower (35% and 20%, respectively) than in the non-treated group (70%). We found that FBRA reduced the number of inflammatory cells infiltrating into the sponge. FBRA administration did not cause myelosuppression, which indicated that the anti-inflammatory effects of FBRA took place at the inflammatory lesion. FBRA did not have antitumor effects on the implanted QRsP-11 tumor cells, which is a tumorigenic cell line established from a tumor arisen after co-implantation of QR-32 cells with sponge. FBRA did not reduce formation of 8-hydroxy-2′-deoxyguanine adducts, a marker of oxidative DNA damage in the inflammatory lesion; however, it reduced expression of inflammation-related genes such as TNF-α, Mac-1, CCL3 and CXCL2. These results suggest that FBRA will be an effective chemopreventive agent against inflammation-related carcinogenesis that acts by inhibiting inflammatory cell infiltration into inflammatory lesions.

## 1. Introduction

Since a possible link between inflammation and carcinogenesis was first indicated by Rudolf Virchow in the 19th century, results from animal and epidemiological studies have supported his hypothesis [1]. It is estimated that inflammation is linked to approximately 20% of all deaths from cancer worldwide [2]. Particularly, chronic inflammation is closely associated with the risk of cancer; for example, inflammatory diseases persisting over decades, such as reflux esophagitis, *Helicobacter pylori* gastritis, inflammatory bowel disease, hepatitis B virus and hepatitis C virus infections trigger esophageal cancer, gastric cancer, colorectal cancer and hepatocellular carcinoma, respectively [1,3].

Because of the obvious consequences of inflammation leading to carcinogenesis, chemoprevention by anti-inflammatory agents has been devised as a preventive strategy against inflammation-related carcinogenesis. It is generally known that steroidal drugs and non-steroidal anti-inflammatory drugs (NSAIDs) exert anti-inflammatory effects by inactivation of cyclooxygenase (COX) and thereafter suppression of prostaglandin synthesis. Epidemiologic studies revealed that the use of NSAIDs protects patients with *Helicobacter pylori* gastritis or ulcerative colitis against cancer development [4,5]. The International Agency for Research on Cancer evaluates that typical NSAIDs such as aspirin and sulindac are chemopreventive agents [6], and that aspirin is particularly effective against colon carcinogenesis [7]. In this regard, NSAIDs are promising chemopreventive drugs; however, use of NSAIDs causes unexpected lethal side effects, such as cardiovascular disease, ulcerating disease, hypertension and acute renal failure in some cases [8]; to avoid them, safe compounds with antiphlogistic effects are needed. Considering the importance of safety in long-term administration, we have investigated natural compounds, ideally to find a candidate agent in foods.

Fermented brown rice and rice bran with *Aspergillus oryzae* (FBRA) is a processed food prepared from brown rice and its bran. Previous studies reported that FBRA has chemopreventive effects against chemical carcinogenesis, including azoxymethene-induced colon cancer [9], diethylnitrosoamine-induced liver cancer [10], *N*-nitrosomethylbenzylamine-induced esophageal cancer [11], *N*-butyl-*N*-(4-hydroxybutyl)-nitrosamine-induced bladder cancer [12], *N*-methyl-*N*′-nitro-*N*-nitrosoguanidine-induced gastric cancer [13], 4-(methylnitrosamino)-1-(3-pyridyl)-1-butanone-induced lung tumor [14], 4-nitroquinoline 1-oxide-induced oral cancer [15] and inflammation-related colon carcinogenesis in a mouse model for human familial adenomatous polyposis [16]. Some of the studies suggested that FBRA inhibited tumor cell proliferation [14], elevation of mRNA expression level of the cytochrome P450 2A5 (Cyp2a5) enzyme that is involved in the mutagenic activation of a carcinogenic compound (nicotine) [14] and expressions of inflammation-related genes [16]. However, no conclusion has been drawn yet.

We have established a mouse model of inflammation-related carcinogenesis; in this model, regressive fibrosarcoma cells (QR-32 cells) grow lethally after being co-implanted subcutaneously with a foreign body, a piece of gelatin sponge to induce inflammation at the site of implantation. Links between inflammation and carcinogenesis are evident from the following four findings: (i) Neutrophils initially and predominantly infiltrate into the inserted sponge and cause foreign-body-induced inflammation [17]. The inflammatory cells separated from the sponge can convert QR-32 cells into tumorigenic ones if they are mixed and implanted in mice [17,18]; (ii) Elimination of neutrophils by administering anti-neutrophil antibody (RB6) reduces the number of mice that form tumors [17]; (iii) Infiltration of neutrophils is abolished and acquisition of tumorigenic phenotypes is suppressed in the integrin-beta-2 (leukocyte adhesion molecule) knockout mice [17]; (iv) Inflammatory-cell-derived reactive oxygen species (ROS) or nitric oxide directly contributes to the inflammation-related carcinogenesis, which was determined in gp91^phox^ (one of the ROS-generating enzymes) knockout mice [19] or by administering an inhibitor for inducible nitric oxide synthase [20].

We therefore used gelatin-sponge-induced inflammation, to investigate the direct association between inflammation and carcinogenesis *in vivo*. By using the model, we found that the chemopreventive agent could suppress the inflammation-related carcinogenic process. We herein report that FBRA reduced the frequency of inflammation-related carcinogenesis through suppression of inflammatory cell infiltration into the inflamed sites by inhibiting the expression of inflammation-related genes.

## 2. Materials and Methods

### 2.1. Cell Lines and Culture Conditions

The origin and characteristics of the QR-32 cells have been described previously [21]. Briefly, a transplantable fibrosarcoma, BMT-11, was induced in a C57BL/6 mouse with 3-methylcholanthrene, and a tumorigenic clone, BMT-11 cl-9, was subsequently isolated by limiting dilution. BMT-11 cl-9 cells were exposed *in vitro* to quercetin, which gave rise to a number of random subclones [21]. They spontaneously regressed when injected into normal syngeneic mice. One of the cell clones, QR-32, was used in this study [22].

QR-32 cells and its derived tumorigenic cell line (QRsP-11) were maintained in Eagle minimum essential medium (05900, Nissui Pharm., Tokyo, Japan) containing 8% fetal bovine serum (1370978, GIBCO); and B16BL6 melanoma cells were maintained in Dulbecco’s modified Eagle’s medium (05919, Nissui Pharm., Tokyo, Japan) with 10% fetal bovine serum. The cell lines were maintained at 37 °C in a humidified 5% CO_2_/95% air mixture.

### 2.2. Mice

C57BL/6 mice (female, 5 weeks old) obtained from Nippon SLC (Hamamatsu, Japan) were maintained under SPF conditions, in light from 7:00 a.m. to 7:00 p.m., at 23 ± 3 °C and 50% ± 10% humidity in the Institute for Animal Experimentation of Tottori University and used after one week acclimatization. Mice were fed with a basal diet (MF, Oriental Yeast Co., Ltd., Tokyo, Japan) alone, or MF supplemented with a processed food prepared by fermenting brown rice and its bran with *Aspergillus oryzae* (FBRA). The FBRA-containing diets had been fed starting 2 days before the implantation and throughout the experiment. The experimental protocol was approved by the Committee of the Institute for Animal Experimentation of Tottori University (14-Y-14).

### 2.3. Inflammation-Related Carcinogenesis Model

Mice were divided randomly into three groups: basal diet, 5% and 10% FBRA-containing diet. After mice were anaesthetized, a small incision was made in the right flank of the pelvic region. A piece of gelatin sponge (10 × 5 × 3 mm; Spongel, Astellas Pharm., Tokyo, Japan) was inserted, and the wound was closed with clips. QR-32 cells (1 × 10^5^ cells/0.1 mL) were then immediately injected into the inserted sponge [18]. Body weight and the tumor diameter were recorded twice weekly. Tumor volume was calculated by the formula as follows: Tumor volume = length × width^2^.

### 2.4. Subcutaneous Tumor Growth

For evaluation of tumor growth, QRsP-11 cells (5 × 10^5^) or B16BL6 cells (1 × 10^6^) were implanted subcutaneously into mice. The mice were sacrificed when they were moribund, for assessing metastasis.

### 2.5. Determination of the Total Numbers and the Cell Types of the Peripheral Blood Leukocytes, Bone Marrow Cells and Gelatin Sponge-Infiltrated Cells

After five days, inserted-sponge was removed and then incubated with 0.2% collagenase at 37 °C for collecting sponge-infiltrated cells. Peripheral blood was collected from a postcaval vein and bone marrow cells were collected from femur of the same mouse. Peripheral blood leukocytes, bone marrow cells and gelatin-sponge-infiltrated cells were counted with a hemacytometer after erythrocyte lysis with Tris-buffered ammonium chloride. We also carried out differential counts of the collected cells stained with May-Grüenwald’s and Giemsa solution (131-12811 and 079-04391, respectively, Wako Pure Chemical Inc., Osaka, Japan).

### 2.6. Immunohistochemistry

Inserted gelatin sponge co-implanted with QR-32 cells were removed and fixed with Bouin’s solution overnight, and immersed sequentially in 50%, 75% and 99% ethanol every 24 h to remove picric acid. The tissue samples were dehydrated, and embedded in paraffin. Sections 4 μm thick were examined with hematoxylin-eosin staining or by immunohistochemistry. The sections were stained by using a Histofine mouse stain kit (414322F, Nichirei, Tokyo, Japan). The primary antibody used in this study was a mouse monoclonal antibody against 8-hydroxy-2′-deoxyguanine (8-OHdG, MOG-100, 10 μg/mL; Japan Institute for the Control of Aging, Shizuoka, Japan). Immunoreactions were visualized with diaminobenzidine and the sections were counterstained with hematoxylin.

### 2.7. Quantification of Immunohistochemically Positive Cells

For quantification of 8-OHdG expressions, we microscopically counted the number of positive infiltrated cells among randomly selected 200 cells in the mounted tissue.

### 2.8. RNA Extraction, cDNA Preparation and Quantitative Real-Time PCR Analysis

Frozen tissue was crushed into powder in a mortar with liquid nitrogen. Total RNA was isolated from the tissue with a TRIzol reagent (GIBCO/BRL, Gaithersburg, MD, USA). cDNA synthesis was performed as described previously [23]. Briefly, three hundred ng of total RNA was used for the synthesis of the first-strand cDNA in a 20 μL reaction mixture containing 1x first-strand buffer, 7.5 mM DTT, 0.5 mM MgCl_2_, 0.5 mM dNTP, 100 pg random primer and Molony murine leukemia virus reverse transcriptase (GIBCO/BRL). PCR amplification of cDNA was performed in a 50 μL reaction mixture containing 1x universal buffer, 200 nM of each primer, 0.2 mM dNTPs and 2.5 units of Taq polymerase (Nippon Gene, Tokyo, Japan). Gene-specific primers were designed to span the noncoding intron region: *Catalase* upstream, 5′-ccttcaagttggttaatgcaga-3′; *Catalase* downstream, 5′-caagtttttgatgccctggt-3′; *CCL3* upstream, 5′-accatgacactctgcaacca-3′; *CCL3* downstream, 5′-gatgaattggcgtggaatct-3′; *CXCL1* upstream, 5′-cttgaaggtgttgccctcag-3′; *CXCL1* downstream, 5′-aagggagcttcagggtcaag-3′; *CXCL2* upstream, 5′-gcgcccagacagaagtcat-3′; *CXCL2* downstream, 5′-tccaggtcagttagccttgc-3′; *CXCR2* upstream, 5′-agttgggagccactctgct-3′; *CXCR2* downstream, 5′-ccaccttgaattctcccatc-3′; *G-CSF* upstream, 5′-cctggagcaagtgaggaaga-3′; *G-CSF* downstream, 5′-ccagcaacaccagctcct-3′; *GM-CSF* upstream, 5′-gggcaatttcaccaaactca-3′; *GM-CSF* downstream, 5′-atgaaatccgcataggtggt-3′; *Gpx1* upstream, 5′-tttcccgtgcaatcagttc-3′; *Gpx1* downstream, 5′-tcggacgtacttgagggaat-3′; *IFN-γ* upstream, 5′-gaggaactggcaaaaggatg-3′; *IFN-γ* downstream, 5′-gctgatggcctgattgtctt-3′; *IL-1β* upstream, 5′-cctcacaagcagagcacaag-3′; *IL-1β* downstream, 5′-tggggaaggcattagaaaca-3′; *IL-2* upstream, 5′-cccacttcaagctccacttc-3′; *IL-2* downstream, 5′-ggagctcctgtaggtccatc-3′; *IL-4* upstream, 5′-tcaacccccagctagttgtc-3′; *IL-4* downstream, 5′-tgtgacctcgttcaaaatgc-3′; *IL-6* upstream, 5′-aagcgagagtccttcagagaga-3′; *IL-6* downstream, 5′-gagcattggaaattggggta-3′; *Mac-1* upstream, 5′-ggctttggacagagtgtggt-3′ *Mac-1* downstream, 5′-agagggcacctgtctggtta-3′; *M-CSF* upstream, 5′-ggctccaggaactctccaat-3′; *M-CSF* downstream, 5′-cagcagctggagaggagtct-3′; *Nox2* upstream, 5′-caagatggaggtgggacagt-3′; *Nox2* downstream, 5′-gcttatcacagccacaagca-3′; *Prdx1* upstream, 5′-gtgagacctgtggctcgac-3′; *Prdx1* downstream, 5′-tgtccatctggcataacagc-3′; *Sod1* upstream, 5′-caggacctcattttaatcctcac-3′; *Sod1* downstream, 5′-tgcccaggtctccaacat-3′; *Sod2* upstream, 5′-gacccattgcaaggaacaa-3′; *Sod2* downstream, 5′-gtagtaagcgtgctcccacac-3′; *TGF-β1* upstream, 5′-attcctggcgttaccttgg-3′; *TGF-β1* downstream, 5′-agccctgtattccgtctcct-3′; TNF-α upstream, 5′-acggcatggatctcaaagac-3′; TNF-α downstream, 5′-agatagcaaatcggctgacg-3′; *XO* upstream, 5′-gtcacgatgacgaggacaac -3′; *XO* downstream, 5′-cttgttctgaaggcggtcat-3′; and *β-actin* upstream, 5′-tgaggagcaccctgtgct-3′; *β-actin* downstream, 5′-acatggctggggtgttgaag-3′. The PCR cycles consisted of 5 min initial denaturation at 95 °C, followed by 40 cycles at 95 °C for 1 min, 60 °C for 1 min and 72 °C for 2 min in a thermal cycler (7900 HT, Applied Biosystems, CA, USA). Fold changes in mRNA levels were calculated by the delta-delta CT method using β-actin as an endogenous control.

### 2.9. Statistical Analysis

The significance of the differences in tumor and metastatic incidences was calculated by *X*^2^ test. For evaluating the differences in the ratio of inflammatory cell types, and those in the number of infiltrated cells, peripheral blood leukocytes, bone marrow cells, survival time, tumor volume, gene expression, Student’s *t*-test was used.

## 3. Results

### 3.1. Suppression of Inflammation-Related Carcinogenesis by Administration of FBRA

We examined the chemopreventive effects of fermented brown rice and its bran with *Aspergillus oryzae* (FBRA) on inflammation-related carcinogenesis in a mouse model. Mice were fed with basal diet, 5% FBRA- or 10% FBRA-containing diet freely starting at day two before co-implantation and throughout the experiment. As a result, QR-32 cells co-implanted with a piece of gelatin sponge grew lethally in 14 out of 20 non-treated mice (70%), seven out of 20 5% FBRA-administered mice (35%), and four out of 20 10% FBRA-administered mice (20%). Namely, the tumor formation was significantly reduced in the FBRA-treated mice compared to that in non-treated mice (Table 1).

**Table 1:** Inhibition of inflammation-related carcinogenesis by administration of FBRA.

Treatment ^a^	Gelatin Sponge Implantation	QR-32 Cells Injection ^b^	No. of Mice with Tumor/No. of Mice Tested (%)		
			Experiment I	Experiment II	Total
None	-	+	0/10	0/10	0/20 (0)
None	+	+	7/10	7/10	14/20 (70)
5% FBRA	+	+	4/10	3/10	7/20 ^c^ (35)
10% FBRA	+	+	2/10^c^	2/10^c^	4/20 ^d^ (20)

^a^ No FBRA, 5% or 10% FBRA containing-diet administration had been started two days before co-implantation and continued throughout the experiment; ^b^ One × 10^5^ QR-32 cells were co-implanted subcutaneously with or without gelatin sponge in mice; ^c^ *p* < 0.05; ^d^ *p* < 0.01 *vs.* basal diet-administered mice co-implanted QR-32 cells with gelatin sponge.

**Figure 1:**
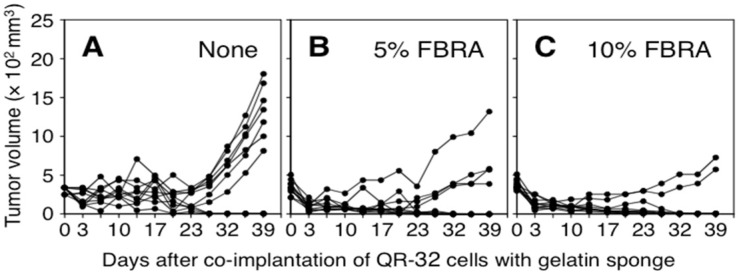
Growth curves of QR-32 cells co-implanted with gelatin sponge in mice. The administration of basal diet (**A**); 5% FBRA (**B**) or 10% FBRA (**C**) started two days before the co-implantation and throughout the experiment. Inflammation-related carcinogenesis was suppressed by FBRA administration.

Growth curves of the arising tumors are shown in Figure 1. Subcutaneous tumor volumes increased immediately after the co-implantation because of the insertion of the sponge. From three days after implantation, the volumes decreased because of absorption of the sponge in the body tissue. Lethal growth of QR-32 cells occurred around 23 days after co-implantation.

### 3.2. Effects of FBRA Administration on Proliferation of Transplantable Tumor Cells

Two possible explanations were posed as to the inhibition of inflammation-related carcinogenesis by FBRA: (1) FBRA directly had cytotoxic effects on the tumor cells converted from QR-32 cells; (2) FBRA suppressed infiltration by inflammatory cells.

**Figure 2:**
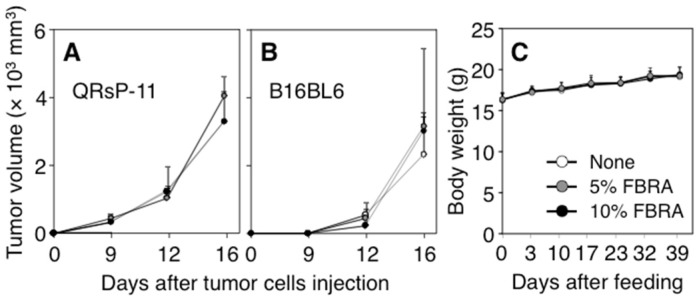
No difference was found in the tumor growth or body weights between basal and FBRA-containing diets. Five × 10^5^ QRsP-11 fibrosarcoma cells (**A**) and 1 × 10^6^ B16BL6 melanoma cells (**B**) were implanted into mice subcutaneously. Body weights of mice co-implanted QR-32 cells with gelatin sponge as shown in Figure 1 remained unchanged after FBRA administration (C).

To assess the first possibility, we examined whether FBRA directly exerts anti-tumor effects on QRsP-11 cells, which were established from an arising tumor in our model [18]. We implanted QRsP-11 tumor cells into mice, and observed no inhibitory effects on the growth of QRsP-11 tumor cells in the 5% or 10% FBRA-fed groups (Figure 2A). A similar result was observed in another cell line, B16BL6 melanoma cells (Figure 2B). Namely, suppressive effects of FBRA were not observed on the growth of tumor cells in either of two different cell lines. Further, FBRA treatment did not alter the incidences of tumor formation, survival period, or metastatic ability compared to those of non-treated mice (Table 2).

**Table 2:** Tumor incidence and metastatic ability of QRsP-11 tumor cells or B16BL6 melanoma cells were not influenced by FBRA administration.

Cells	Subcutaneous Tumorigenicity ^a^			Spontaneous Metastasis ^c^		
	Treatment ^b^	No. of Mice with Tumor/No. of Mice Tested (%)	Mean Survival Time (Days)	No. of Mice with Lung Metastasis/No. of Mice Tested (%)	No. of Mice with Lymph Node (LN) Metastasis/No. of Mice Tested (%)	
					Inguinal LN	Axillary LN
QRsP-11	None	5/5 (100)	34 ± 6	0/5 (0)	0/5 (0)	0/5 (0)
	5% FBRA	5/5 (100)	33 ± 10	0/5 (0)	0/5 (0)	0/5 (0)
	10% FBRA	4/4 (100)	25 ± 14	0/5 (0)	0/5 (0)	0/5 (0)
B16BL6	None	5/5 (100)	26 ± 4	2/5 (40)	0/5 (0)	0/5 (0)
	5% FBRA	5/5 (100)	27 ± 1	0/5 (0)	1/5 (20)	0/5 (0)
	10% FBRA	5/5 (100)	28 ± 4	1/5 (20)	1/5 (20)	2/5 (40)

^a^ Five × 10^5^ QRsP-11 tumor cells or 1 × 10^6^ B16BL6 melanoma cells were implanted subcutaneously in mice; ^b^ No FBRA- or FBRA-containing diet administration was started two days before implantation and continued throughout the experiment; ^c^ The tumor-bearing mice were sacrificed when they were moribund. Lung and lymph node metastatic incidences were evaluated macroscopically.

The administration of 5% or 10% FBRA (0.1 or 0.3 g/day/mouse, respectively) did not cause obvious side effects, such as weight loss or alteration in the appearance or behavior of the tumor-bearing mice during the observation period. Data of the average body weight are shown in Figure 2C.

### 3.3. Inhibition of Infiltration of Inflammatory Cells into Gelatin Sponge by FBRA

**Figure 3:**
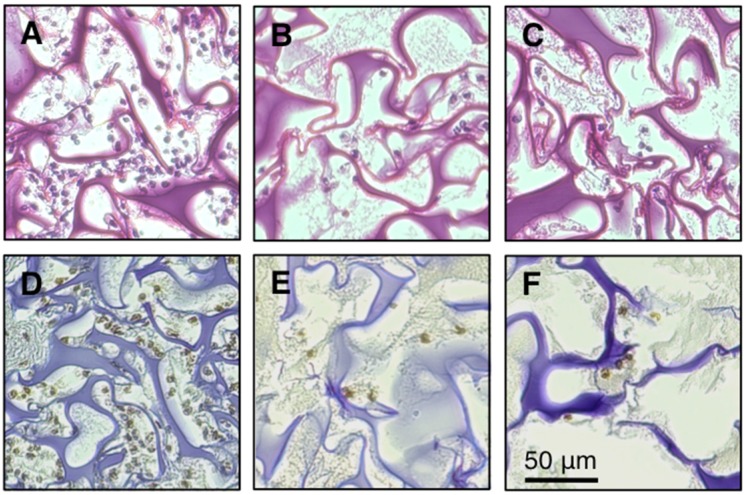
H & E staining and immunohistochemical detection of 8-OHdG at the inflammatory site. Histological sections were obtained from the mice five days after co-implantation of QR-32 cells with gelatin sponge. (**A**,**D**), tissues from basal diet group; (**B**,**E**), from 5% FBRA group; (**C**,**F**), from 10% FBRA group; (**A**–**C**) were stained with H & E. Immunohistochemistry was performed with antibody against 8-OHdG (**D**–**F**). Scale bar, 50 μm.

We next assessed the second possibility, whether FBRA administration inhibits infiltration of inflammatory cells into sponge, by histological and quantitative analyses [23,24]. A piece of sponge was inserted into a mouse subcutaneously two days after the start of FBRA feeding, and removed five days after the insertion for examination. Figure 3A shows that numerous inflammatory cells infiltrated into the sponge in the non-treated group. However, the infiltration was inhibited markedly in FBRA-fed groups (Figure 3B,C).

We then evaluated the effects of FBRA on infiltration of inflammatory cells by counting the infiltrated cells after digesting the sponge with collagenase. Administration of FBRA significantly reduced the numbers of infiltrated cells (Table 3).

**Table 3:** Inhibition of infiltration of inflammatory cells into gelatin sponge by FBRA administration.

Treatment ^a^	Co-Implantation of QR-32 Cells with Gelatin Sponge ^b^	No. of Gelatin Sponge-Infiltrated Cells (× 10^4^) ^c^	No. of Peripheral Blood Leukocytes (× 10^2^/μL)	No. of Bone Marrow Cells (× 10^6^)
None	-	NA ^d^	18 ± 4	17 ± 2
None	+	143 ± 25	22 ± 6	20 ± 4
5% FBRA	+	94 ± 24 ^e^	26 ± 4	16 ± 5
10% FBRA	+	86 ± 22 ^e^	20 ± 5	18 ± 4

^a^ Basal or FBRA-containing diet had been started two days before co-implantation procedure and continued throughout the experiment; ^b^ One × 10^5^ QR-32 cells were injected into pre-inserted gelatin sponge; ^c^ The sponge was removed and digested in the collagenase solution. The sponge-infiltrated cells were then collected and counted; ^d^ NA, not applicable; ^e^ *p* < 0.001 *vs.* basal diet-administered mice. Each data represents the results of three independent experiments.

### 3.4. Timing of FBRA Administration for Suppressing the Infiltration of Inflammatory Cells

We examined the administration time and period (days) when FBRA could exert inhibitory effects on the inflammatory cell infiltration. Mice were separately fed with FBRA starting (i) before and (ii) after the implantation of gelatin sponge. A decrease of the number of infiltrated cells was confirmed in the mice fed with FBRA starting at least three days before the implantation; in the group fed with FBRA starting only one day before the implantation, no effect was detected. And FBRA administration starting after the implantation had no inhibitory effects (Table 4).

**Table 4:** Timing of FBRA administration for suppressing the infiltration of inflammatory cells.

Treatment Period	No. of Gelatin Sponge-Infiltrated Cells (× 10^4^) ^a^		
	None	5% FBRA	10% FBRA
Day −2–5	215 ± 15	146 ± 21 ^b^	134 ± 21 ^b^
Day 0–1	212 ± 35	200 ± 14	204 ± 19
Day 0–3	261 ± 16	244 ± 31	245 ± 18
Day 0–5	202 ± 30	215 ± 60	230 ± 30
Day −1–0	233 ± 25	227 ± 26	218 ± 15
Day −3–0	277 ± 15	145 ± 6 ^b^	140 ± 16 ^b^
Day −5–0	238 ± 26	138 ± 32 ^b^	152 ± 27 ^b^

^a^ Five days after implantation of gelatin sponge, sponge-infiltrated cells were collected and counted; ^b^ *p* < 0.01 *vs.* basal diet-administered mice. Data represent the results of at least two independent experiments.

### 3.5. Types of Inflammatory Cells Affected by FBRA Administration

To examine whether the inhibition of infiltration of inflammatory cells was due to systemic immunosuppression in FBRA-fed mice, we assessed presence or absence of myelosuppression. After removing the femur from the FBRA-administered mice, we counted the total number of bone marrow cells. At the same time, we collected peripheral blood samples and counted the number of leukocytes. We found that neither the number of bone marrow cells nor that of peripheral blood leukocytes was affected by FBRA feeding (Table 3). These results demonstrated that the inhibitory effect of FBRA on cell infiltration was neither due to systemic myelosuppression nor immunosuppression.

Next, we examined whether FBRA inhibits certain subtypes of leukocytes. To compare the types of the inflammatory cells, we collected gelatin-sponge-infiltrating cells, peripheral blood leukocytes and bone marrow cells from the mice fed with or without FBRA. We found that percentages of monocytes/macrophages, granulocytes and lymphocytes in those cells were not affected by FBRA ingestion (Table 5).

**Table 5:** Differences in leukocyte counts in mice with administration of FBRA.

Cell Source ^a^	Treatment ^b^	Co-Implantation of QR-32 Cells with Gelatin Sponge ^c^	Differential Leukocyte Counts (%) ^d^		
			Monocytes/Macrophages	Granulocytes	Lymphocytes
Gelatin sponge-Infiltrated	None	+	11 ± 2	57 ± 3	32 ± 5
	5% FBRA	+	11 ± 3	59 ± 4	30 ± 1
	10% FBRA	+	11 ± 1	59 ± 2	31 ± 3
Peripheral blood	None	-	2 ± 1	18 ± 4	81 ± 4
	None	+	2 ± 1	16 ± 4	82 ± 4
	5% FBRA	+	3 ± 1	16 ± 6	81 ± 5
	10% FBRA	+	3 ± 0	17 ± 7	81 ± 7
Bone marrow	None	-	5 ± 2	72 ± 5	23 ± 3
	None	+	5 ± 1	73 ± 2	22 ± 2
	5% FBRA	+	5 ± 1	71 ± 3	24 ± 3
	10% FBRA	+	5 ± 1	72 ± 3	23 ± 2

^a^ Five days after co-implantation, inflammatory cells infiltrated into gelatin sponge, peripheral blood leukocytes and bone marrow cells were collected and counted; ^b^ No FBRA- or FBRA-containing diet had been started two days before co-implantation and continued throughout the experiment; ^c^ One × 10^5^ QR-32 cells were co-implanted with gelatin sponge; ^d^ Differential counts in smear preparations of the collected cells stained with May-Grüenwald’s and Giemsa solution. Data represent the results of two or three independent experiments.

### 3.6. Effects of FBRA Administration on the Ability to Induce Oxidative Stress

**Figure 4:**
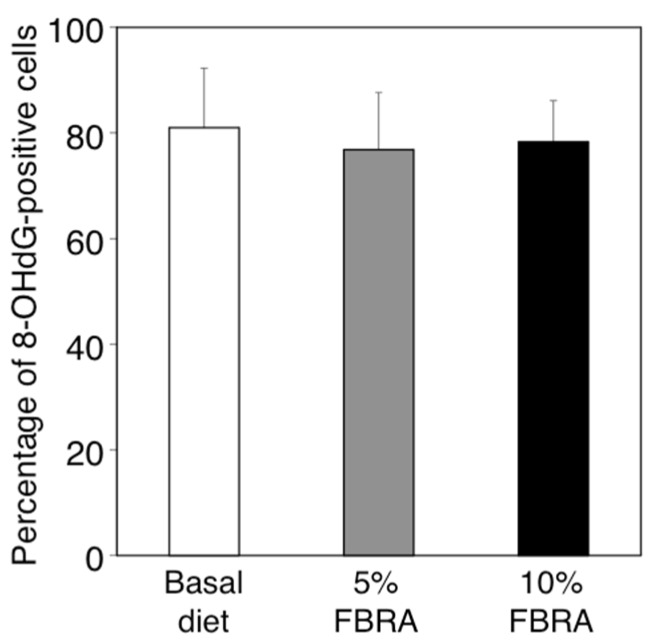
No difference was found between basal and FBRA-containing diets in 8-OHdG formation in the tissues inflamed by co-implantation of gelatin sponge with QR-32 cells. Bar graphs show means ± SD (*n* = 3 in each group).

Using our model, we have observed that excess amounts of ROS are produced by inflammatory cells infiltrating into sponge and lead to tumorigenic conversion of QR-32 cells [19]. We therefore explored the effects of FBRA administration on oxidative stress at the inflamed site. The degree of oxidative stress was evaluated immunohistochemically by formation of 8-OHdG, a marker of oxidative DNA damage (Figure 3D–F). The percentage of 8-OHdG positive cells against the total infiltrating cells was found to be about 80% in each group, showing no difference among the three groups (Figure 4).

### 3.7. Changes in the Expressions of Inflammation-Related Genes at the Inflammatory Site by Administration of FBRA

**Figure 5:**
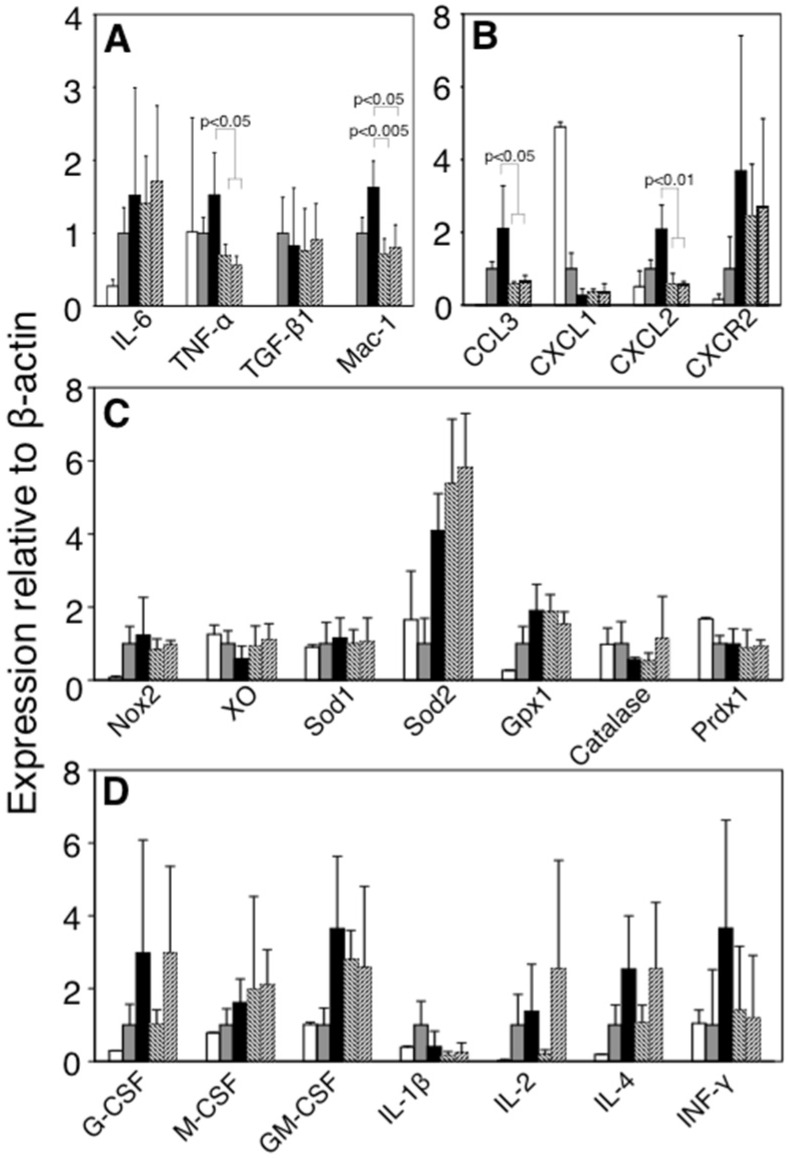
Alteration of inflammation-related gene expressions of inflammatory cells and hematopoietic regulatory factors of bone marrow cells after administration of FBRA. Real-time RT-PCR analysis was performed to quantify the changes in mRNA expressions of cytokines and leukocyte adhesion molecules (**A**); chemokines (**B**) and oxidative stress-related molecules (**C**) in the inflammatory lesions, and interleukins/growth factors (**D**) in the bone marrow cells after administration of basal diet (closed bar), 5% (diagonal right down bar) or 10% FBRA containing diet (diagonal right up bar). For the control, inflammatory cells alone (with implantation of gelatin sponge only, gray bar) and cultured QR-32 cells (open bar) were used. Bar graphs show means ± SD (*n* = 4 in each group). Significant differences were observed as compared to those of basal diet group.

To clarify the mechanism responsible for inhibiting the infiltration of inflammatory cells under FBRA treatment, we compared the expressions of inflammation-related molecules of the infiltrated cells. TNF-α, a pro-inflammatory cytokine, and Mac-1, an adhesion molecule, were significantly down-regulated under FBRA administration (Figure 5A). Moreover, CCL3, a monocyte attractant, and CXCL2, a neutrophil attractant, were also down-regulated (Figure 5B). On the other hand, expressions of ROS-relating enzymes, such as gp91^phox^ (Nox2) and xanthine oxidase (XO), and antioxidative enzymes, such as copper zinc superoxide dismutase (Sod1), manganese superoxide dismutase (Sod2), glutathione peroxidase 1 (Gpx1), catalase and peroxiredoxin1 (Prdx1) were not different between basal diet-treated group and FBRA-treated group (Figure 5C).

### 3.8. No Change in Gene Expression of Bone Marrow Cells by FBRA

To confirm whether FBRA treatment affects the myeloid cells or not, we assessed the expressions of cytokines and growth factors in the bone marrow cells, and found that FBRA feeding did not affect those expression levels (Figure 5D).

## 4. Discussion

In this study, we found that oral administration of FBRA, brown rice and its bran fermented with *Aspergillus oryzae*, prevented inflammation-related carcinogenesis by inhibiting infiltration of inflammatory cells.

Previous reports demonstrated that administration of FBRA exerts preventive effects on chemical carcinogenesis induced in the oral cavity, lung, esophagus, stomach, liver, colon and bladder [9,10,11,12,13,14,15]. FBRA also has suppressive effects on inflammation-related carcinogenesis such as intestinal tumor formation induced by administration of dextran sodium sulfate in *Apc*^Min/+^ mice, which have a germline mutation in the *Apc* gene responsible for familial adenomatous polyposis [16,25].

We used a mouse model of carcinogenesis in which inflammation was induced by implantation of a foreign body, instead of using a prophlogistic chemical agent. The advantage of this model is that quantitative evaluation of the inflammatory reaction is possible, because the number of infiltrated cells into the foreign body is directly countable. And the cell types and gene expression of the inflammatory cells harvested from the foreign body can also be analyzed. By measuring the numbers of the peripheral blood leukocytes and bone marrow cells from the mice which showed decreased inflammatory responses, we found that the counts and types of the cells obtained from FBRA-fed mice were not different, compared to those of the basal diet-treated mice (Table 3). Additionally, we found that FBRA inhibited inflammatory cell infiltration as a whole, instead of certain subtypes of leukocytes specifically (Table 3 and Table 5). From these results, we suggest that the anti-inflammatory effects of FBRA are not due to systemic immunosupression but more likely by modulation of the master regulating factor(s) that control the expression of adhesion molecules and chemokines at an inflamed site.

Since inflammatory cells roll through vascular endothelial cells, adhere to the endothelium and then extravasate into inflammatory tissues, both cell adhesion molecules and chemokines must be important factors for their infiltration [26]. Mac-1, an adhesion molecule, and CCL3 and CXCL2, leukocyte-attractant chemokines, were down-regulated by FBRA administration. Generally these three molecules are known to be up-regulated, following typical inflammatory reaction [27,28,29], and more importantly, regulated by TNF-α signaling [30,31,32,33]. Since qRT-PCR analyses showed that FBRA decreased the expression of TNF-α in the inflamed site (Figure 5), we assume that FBRA suppresses the expression of TNF-α, a master molecule, and subsequently those of Mac-1, CCL3 and CXCL2 which are in the downstream pathways of TNF-α. We are currently investigating whether TNF-α has a role in the mechanism by using TNF-αknockout mice.

Taking the analysis results of our inflammation-related carcinogenesis model into consideration, we assumed four possible mechanisms/hypotheses for the suppression of carcinogenesis by FBRA ingestion: (i) By reduction of infiltration of inflammatory cells (17); (ii) By suppressing the production of ROS/nitric oxide derived from inflammatory cells [19,20], even if infiltration of inflammatory cells is not suppressed; (iii) By elimination of inflammatory-cells-derived ROS through antioxidative enzymes induced at inflamed lesions [34]; and (iv) By specific cytotoxicity to the malignantly converted QR-32 cells by inflammation. We assessed these four possibilities as to the results in this study and concluded that the inhibitory effect of FBRA was likely by mechanism (i) (Figure 3 and Table 3). FBRA did not have an effect in suppressing oxidative stress (8-OHdG formation), and expression of Nox2 and XO, both ROS-relating enzymes, were not altered by FBRA administration, which excluded the possibility of (ii) (Figure 3, Figure 4 and Figure 5). The hypothesis (iii) was also excluded because expressions of major antioxidative enzymes (Sod1, Sod2, Gpx1, catalase and Prdx1) were not enhanced by FBRA (Figure 5). The possibility of (iv) was thought to be low because FBRA did not inhibit growth of implanted tumor cells (QRsP-11 and B16BL6; Figure 2 and Table 2).

Interestingly, we did not detect a decrease of peripheral blood leukocytes or bone marrow cells, or abnormal differentiation of these cells due to administration of FBRA (Table 3 and Table 5). Moreover, no adverse effect of FBRA such as weight reduction was observed (Figure 2C) in this study, or in other animal experiments or a clinical trial [13,35]. Possibility of adversity such as side effects is thought to be low because FBRA is derived from natural compounds. By further confirming the safety of detailed mechanisms of FBRA, it would be possible to apply it not only to prevention of inflammation-related carcinogenesis but also to prevention of excess inflammatory or immune reactions.

## 5. Conclusions

Considering the close link between inflammation and carcinogenesis, side-effect-free agents are needed to prevent/treat inflammation, since steroidal drugs and non-steroid anti-inflammatory drugs (NSAIDs) often accompany unfavorable adverse effects when used for controlling persistent inflammatory diseases. We revealed that oral administration of food-borne FBRA, brown rice and its bran fermented with *Aspergillus oryzae*, prevented inflammation-related carcinogenesis by suppressing inflammatory cell infiltration into the inflamed sites but not inducing myelosuppression. Under the treatment with FBRA, expressions of inflammation-related genes such as TNF-α, Mac-1, CCL3 and CXCL2 were down-regulated at the inflammatory lesion. These findings suggest that FBRA is a potent agent for chemoprevention against inflammation-related carcinogenesis.
